# The E2F4 prognostic signature is also predictive of the pathological response of breast cancer to chemotherapy

**DOI:** 10.1186/s13058-015-0559-2

**Published:** 2015-04-10

**Authors:** François Bertucci, Pascal Finetti, Daniel Birnbaum

**Affiliations:** Département d’Oncologie Moléculaire, Centre de Recherche en Cancérologie de Marseille, Institut Paoli-Calmettes, UMR1068 Inserm, 232 boulevard de Sainte-Marguerite, 13009 Marseille, France; Département d’Oncologie Médicale, Centre de Recherche en Cancérologie de Marseille, Institut Paoli-Calmettes, UMR1068 Inserm, 232 boulevard de Sainte-Marguerite, 13009 Marseille, France; Faculté de Médecine, 13385 Aix-Marseille Université, 27 boulevard Jean Moulin, Marseille cedex 5, France

We read with interest the article by Khaleel and colleagues reporting a new prognostic signature in hormone receptor (HR)-positive breast cancer based on mRNA expression of target genes of the E2F4 transcription factor [1]. The clinical relevance comes from its independent prognostic value and its biological significance (mainly regulation of cell cycle and proliferation, reflecting high E2F4 activity). When compared with patients with low score for the signature (low risk), patients with high score (high risk) showed shorter relapse-free survival, making them candidates for adjuvant chemotherapy. Whereas adjuvant chemotherapy is recommended for most human epidermal growth factor receptor 2 (HER2)-positive or triple-negative tumors, indications are more challenging for HR^+^/HER2^−^ tumors, which are candidates for either adjuvant hormone therapy alone or both hormone therapy and chemotherapy.

We wondered whether high-risk HR^+^/HER2^−^ tumors were more chemosensitive than low-risk HR^+^/HER2^−^ tumors. We gathered gene expression data for 1,247 breast cancers treated with neoadjuvant anthracycline-based chemotherapy and with available pathological response (Additional file 1), pathological complete response (pCR) being defined as no residual invasive cancer in the breast and axillary lymph nodes. All cases were assigned a relapse risk according to the metagene based on average expression of 199 E2F4 target genes in each standardized dataset. We analyzed the predictive value of the E2F4 metagene for the pathological response to chemotherapy in the 582 HR^+^/HER2^−^ tumors (12% pCR rate). High-risk tumors were associated (Additional file 2) with ductal type, grade 3, and higher pCR rate, which was 17% versus 8% in the low-risk tumors (*P* <0.001). As expected, grade 3 was also associated with higher pCR rate (Table 1). In multivariate analysis, the E2F4 metagene remained predictive for pCR (*P* = 0.027), whereas grade did not. Interestingly, mRNA expression of *E2F4* itself did not predict for the response to chemotherapy, demonstrating the interest of the metagene as a better indicator of E2F4 function than *E2F4* expression level alone.

**Table 1:** **Univariate and multivariate analyses for pathological complete response**

	**Univariate analysis**			**Multivariate analysis**		
	***n***	**Odds ratio (95% CI)**	***P*** **value**	***n***	**Odds ratio (95% CI)**	***P*** **value**
Age, >50 years versus ≤50 years	582	0.79 (0.51 to 1.21)	0.37			
Histological type						
ILC versus IDC	240	2.60 (0.71 to 8.12)	0.19			
Other versus IDC		1.09 (0.49 to 2.26)	0.85			
Clinical tumor size, cT2 to cT4 versus cT1	580	0.88 (0.44 to 2.01)	0.79			
Clinical axillary lymph node status, cN1 to cN3 versus cN0	571	1.37 (0.86 to 2.23)	0.27			
Grade						
2 versus 1	529	1.32 (0.43 to 5.98)	0.72	529	1.22 (0.40 to 5.52)	0.80
3 versus 1		5.56 (1.93 to 24.4)	**2.09E-02**		4.37 (1.48 to 19.4)	0.05
E2F4 metagene-based classification, high risk versus low risk	582	2.40 (1.57 to 3.72)	**7.98E-04**	529	1.86 (1.14 to 3.08)	**4.02E-02**

CI, confidence interval; IDC, invasive ductal cancer; ILC, invasive lobular cancer. Bold data indicate significance.

HR^+^/HER2^−^ tumors with an E2F4 high-risk signature were more sensitive to anthracycline-based chemotherapy than low-risk tumors, as already reported with other prognostic signatures [2,3] – including in the adjuvant setting [4,5], where high-risk tumors showed greater benefit from chemotherapy than low-risk tumors. The next step will be to test, retrospectively in randomized prospective trials of adjuvant chemotherapy, the hypothesis that the difference in relapse between patients treated with chemotherapy and untreated patients is larger in the high-risk group than in the low-risk group. Interest in the E2F4 signature is probably higher than expected.

## Additional files

Additional file 1: Table S1. Presenting a description of the eight public breast cancer datasets. Additional file 2: Table S2. Presenting the E2F4 metagene-based classification and clinicopathological correlations.

## Abbreviations

HER2: Human epidermal growth factor receptor 2
HR: Hormone receptor
pCR: Pathological complete response
